# Remote‐Based Brain Endurance Training Enhances Bench Press, Preacher Curl, and Jump Squat Performance in Recreational Athletes

**DOI:** 10.1002/ejsc.70173

**Published:** 2026-04-13

**Authors:** Alexandru Rautu, Josh Woods, Hannah Mortimer, Neil Dallaway, Christopher Ring

**Affiliations:** ^1^ School of Sport, Exercise & Rehabilitation Sciences University of Birmingham Birmingham UK

## Abstract

Supervised laboratory‐based brain endurance training (BET), a form of combined cognitive and exercise training, benefits exercise performance. Given that athletes often train unsupervised, we compared effects of unsupervised remote‐based BET and standard exercise training (ET) on muscular endurance exercise performance. In a pre‐test/training/post‐test design, 22 adults completed 2 laboratory testing and 12 remote‐based gym training sessions. In each laboratory testing session, they performed bench press, preacher curl, and squat jump repetitions to failure. Ratings of perceived exertion (RPE) were obtained after performing 10 bench press and preacher curl repetitions. Participants were randomly assigned to BET (*n* = 10) or ET (*n* = 12) groups. In each remote training session, they performed upper body and core exercises. The BET group completed cognitive tasks whereas the ET group relaxed before/after exercise. From pre‐test to post‐test, the BET group increased repetitions to failure for total (+50%), bench press (+33%), preacher curl (+93%), and squat jump (+28%) repetitions whereas the ET group only increased repetitions for the total (+13%) but did not increase bench press (+13%), preacher curl (+30%), or squat jump (+12%) repetitions. These training‐related improvements in total and squat jump repetitions were greater for the BET group than the exercise group. Moreover, from pre‐test to post‐test, the BET group reported lower RPE for bench press (−22%) and preacher curl (−12%) while the ET group did not change RPE for bench press (−9%) or preacher curl (−3%). In conclusion, remote‐based BET improved muscular endurance performance and reduced perceived effort more than standard training in experienced recreational athletes.

## Highlights

- Brain endurance training (BET), a form of combined cognitive and exercise training, improved total resistance exercise training volume more than standard exercise training alone.
- BET is a flexible training method that can be individually tailored to an athlete's situational demands and constraints in supervised and unsupervised settings using smart mobile technology.
- An emerging body of evidence supports the performance benefits associated with BET, however, bespoke optimization protocols are likely to help elite athletes achieve peak performance.

## 1 Introduction

The psychobiological model of endurance exercise (S. M. Marcora 2008; S. Marcora 2019; S. M. Marcora and Staiano 2010) provides a framework to explain the contribution of psychological factors in the decision to stop exercising. Extending the psychological intensity of motivation model (Brehm and Self 1989) to an exercise context, the psychobiological model proposes that exercise stops when ongoing perceived effort (i.e., intensity of motivation) reaches the point of maximum tolerable effort (i.e., potential motivation). Thus, exercise performance can be extended in two ways. The first way is to increase the maximal tolerable effort further into the physiological reserve between current performance (e.g., personal best) and maximal possible performance (e.g., physiological limit to human performance). The provision of incentives, such as a reward for winning a competition, improves endurance in this way. The second way is to decrease ongoing perceived effort and thereby reach the point of maximal tolerable effort later during exercise. The physiological adaptations that accrue following completion of a standard exercise training (ET) program improve endurance in this way. S. Marcora (2019) reasoned that the relationship between ongoing perceived effort and actual physical effort (i.e., workload) could also be recalibrated by repeatedly exposing athletes to perceptually harder than normal exercise by requiring them to exercise while they performed cognitively demanding tasks that increased ongoing perception of effort. He proposed that repeatedly performing cognitive tasks concurrently while exercising, a training method known as brain endurance training (BET), would subsequently reduce perception of effort during exercise and thereby improve endurance exercise performance (S. M. Marcora et al. 2014). Recent years have witnessed the accumulation of a body of empirical evidence in support of this reasoning (for review see Joseph et al. 2026).

Research studies have established that BET reliably improves exercise performance more than standard ET alone. Specifically, repeatedly performing classic cognitive tasks before, during, and after bouts of exercise (i.e., BET) enhances subsequent exercise performance compared to separate ET alone. Specifically, studies in active young adults confirm that BET enhances aerobic (Barzegarpoor et al. 2021; Staiano et al. 2022, 2023), and muscular (Dallaway et al. 2021; Dallaway et al. 2023, 2024; Díaz‐García et al. 2024) endurance exercise performance more than separate ET. Moreover, there is preliminary evidence that BET also recalibrates the relationship between perceived effort and workload, thereby making exercise at the same exercise intensity feel easier to perform (Barzegarpoor et al. 2021; Staiano et al. 2023). Taken together, previous studies consistently show that BET enhances exercise performance and broadly supports the premise of the psychobiological model (S. M. Marcora 2008; S. Marcora 2019).

A closer examination of the previous studies (Díaz‐García et al. 2024, 2025) that have examined the effects of BET on resistance endurance exercise performance indicates that the benefits of BET were evident when the tests were performed in a state of increased cognitive (and physical) fatigue but not in a fresh state (i.e., no previous cognitive and exercise tasks). It is likely that methodological differences in the experimental designs of the training protocols can help understanding of these findings. Below we first describe the studies and then look for a methodological explanation for the divergent findings.

The first study tested active young adults (Díaz‐García et al. 2024). In each training session (5 sessions per week for 6 weeks), experienced weightlifters performed a series of low intensity cognitive tasks (3‐min incongruent color‐word Stroop; 27‐min per session) intermixed with low intensity resistance (four sets of bench press at 34% 1RM to failure) and plyometric (four sets of squat jumps to failure) exercises. In each testing session, they performed bench press at 34% 1RM (and squat jumps) to failure when fresh and when fatigued (after completing a 30‐min Stroop task). Three (six) weeks of BET improved bench press endurance by 10% (14%) when fresh and 25% (35%) when fatigued. Compared to the ET group, the BET group performed more bench presses when tested after the 30‐min Stroop task after 3 and 6 weeks of training. The second study tested sedentary older adults (Díaz‐García et al. 2025). In each training session (3 sessions per week for 8 weeks), the older adults performed a long low intensity cognitive task (20‐min Stroop) followed by low intensity resistance (four sets of biceps curls with 1 kg weight for 1 min), plyometric (four sets of squats for 1 min) and aerobic (walking for 25 min) exercises. In each testing session, they performed as many repetitions as possible of biceps curls in 30 s (and squats in 30 s and walk in 6 min) when fresh and when fatigued (after completing a 30‐min Stroop task). Four (eight) weeks of BET improved bicep curl endurance by 7% (15%) when fresh and by 12% (13%) when fatigued. Compared to the ET group, the BET group performed more bicep curls when tested after the 30‐min Stroop task following 4 and 8 weeks of training. Based on the methodological features of these studies, it seems likely that the lack of group differences in performance when tested in a fresh state can be explained by the relatively low intensity of the cognitive and/or physical training loads.

This underloading explanation is compatible with the findings of a study of high intensity BET in experienced weightlifters (Rautu et al. 2025). In each training session (2 sessions per week for 4 weeks), experienced weightlifters performed a series of high intensity cognitive tasks (5‐min time‐load dual back and color multi‐source interference; 25‐min per session) intermixed with high intensity resistance exercise (five sets of five repetitions of bench press at 80% 1RM). In each testing session, they performed a 1RM strength test followed by an endurance test where they completed as many repetitions as possible of bench press at 50% 1RM to failure. Four weeks of BET improved bench press endurance by 22%. It is worth noting that this pilot study did not include an ET group and therefore the improvements in performance observed following combined training cannot be interpreted easily.

Nonetheless, the findings of the abovementioned resistance training studies suggest that the benefits of BET are greater with higher cognitive and/or exercise training loads. Accordingly, the current study sought to explore the effects on muscular endurance of 4 weeks of high intensity BET, comprising a series of 3‐min cognitive tasks (2‐back, time‐load dual back, multi‐source interference, switch stop visual; see Dallaway et al. 2024; Rautu et al. 2025) that were intermixed with a program of multiple exercise tasks. In each training session, participants completed 33‐min of demanding cognitive tasks and approximately 60‐min of exercise tasks.

In nearly all of the previous studies showing that BET improves performance more than ET, participants trained under the supervision of a researcher in standardized laboratory or gym settings (e.g., Barzegarpoor et al. 2021; Dallaway et al. 2021; Dallaway et al. 2023; Díaz‐García et al. 2024; Staiano et al. 2022, 2023). A couple of recent studies showed that BET also improved performance more than ET when individuals trained unsupervised: participants completed 4 weeks of unsupervised calisthenic exercises in their own home (Study 1, Dallaway et al. 2024) or gym (Study 2, Dallaway et al. 2024). Taken together, these findings show that both supervised and unsupervised BET can benefit performance. Importantly, all of the previous BET and muscular endurance studies were conducted under direct supervision in laboratory settings. Because athletes often, if not normally, train on their own in unsupervised gym sessions, the current study required participants to train on their own in unsupervised remote gym‐based environments. We expected that this form of unsupervised training would be more practical for future adoption if successful. Accordingly, participants in the present study were provided with a training program to complete remotely in their own gym and at their own time of choosing. Each week comprised a push exercise session (day 1), a pull exercise session (day 4), and a combined push and pull plus core exercise session (day 6).

Our study purposes were twofold. First, we investigated whether BET improves muscular endurance compared to separate ET (control). We hypothesized that BET, with executive function (updating, inhibiting, switching) cognitive tasks performed before, between and after resistance and core exercises, would improve upper‐body resistance exercise (bench press, preacher curl) and lower‐body plyometric exercise (squat jump) performance to failure compared to ET alone. These exercises were chosen as they are commonly studied by researchers and practiced by athletes (who will have acquired basic neural control of the movements). Second, we examined whether BET changes the perception of effort during exercise. We hypothesized that BET would reduce effort perception during bench press and preacher curl exercises compared to ET alone.

## 2 Methods

### 2.1 Participants

Twenty‐two (14 males, 8 females) university students were recruited. Their mean (SD) age was 21.14 (1.25) years and body mass 72.16 (9.83) kg. The estimated bench press one repetition maximum (1RM_e_) was 52.73 (21.72) kg and preacher curl 1RM_e_ was 23.00 (9.87) kg. Each 1RM_e_ was computed with an online calculator (https://strengthlevel.com/) that uses normative strength standard tables (Kilgore 2023) of a person's sex, age, weight, and experience (training status). The inclusion criteria included at least 1 year of weightlifting experience, current gym membership (needed for the remote‐based physical training) and possessing an iPhone (needed for the cognitive training). They were instructed to discontinue their normal training regimen and follow the prescribed training regimen during the study. Before each session, they were asked to sleep at least 7 hours the night before and to refrain from alcohol for 24 hours and strenuous exercise for 48 hours. They were also asked to maintain their diet and avoid supplements. The study protocol (SPP2223‐04) was approved by the University of Birmingham ethics committee. Participants were provided with an information sheet (that described the study protocol, risks, and consequences for withdrawal) and gave written informed consent in accordance with the Declaration of Helsinki. We did explain the broad study purposes (i.e., to examine the effects of training on exercise performance) but did not explain the specific study hypotheses. Power calculations using GPower (Faul et al. 2007) indicated that with a sample size of 22, the study was powered at 80% to detect significant (*p* < 0.05) small‐to‐medium (*f* = 0.31, *η*_*p*_^2^ = 0.09) group by time interaction effects by analysis of variance (Cohen 1992). Past studies have found exercise performance benefits of BET relative to ET with similar sample sizes; 20 (Barzegarpoor et al. 2021), 22 (Staiano et al. 2022), and 24 (Dallaway et al. 2023) participants.

### 2.2 Experimental Design

The study employed a pre‐test/training/post‐test design, with one between‐participant factor (group: BET, ET) and one within‐participant factor (test: pre‐test, post‐test). In this single blinded design, participants were randomly allocated to BET (*n* = 10) or ET (*n* = 12) groups. Participants completed 14 sessions over 6 weeks, comprising a pre‐test (week 1), 12 training sessions (weeks 2–5), and a post‐test (week 6). During the training intervention, they completed 3 training sessions per week for 4 weeks. In each remote gym‐based training session, all participants performed the same exercises, however, during the short breaks before and after exercise sets, the BET group performed brief cognitive tasks whereas the ET group rested. The detailed training protocol is described in Supporting Information S1. Participants were tested in weeks 1 and 6. In each laboratory testing session, they performed bench press, preacher curls, and squat jump exercises. The number of repetitions of these exercises to failure acted as a measure of muscular endurance exercise performance.

### 2.3 Exercise Tests

Participants watched video tutorials and read written explanations about each exercise from the My PT Hub app (https://www.mypthub.net). They also received verbal guidance from the researcher who was an experienced personal trainer. The chosen exercises imposed different demands: bench press uses a large group of upper body muscles and requires a variety of stabilizers to move a greater weight through the sagittal plane, preacher (biceps) curl is an isolation movement targeting a relatively small upper body muscle group that requires few stabilizers, and squat jumps use lower body muscles as well as the core. It is also worth noting that the training program focused on upper body exercises, and, therefore, squat jump performance allowed us to explore any generalization of training (see Dallaway et al. 2024, Study 2). Moreover, all three exercises have been examined in previous BET studies (Dallaway et al. 2024; Díaz‐García et al. 2024, 2025; Rautu et al. 2025). Details of each exercise are provided below.

The flat bench press is a compound (pectoral, deltoid, and triceps muscles) upper body exercise. Participants laid with their back on the bench parallel to the floor. From the starting position (arms extended with hands gripping the bar slightly wider than their shoulders and the weight directly over them), they lowered the 20 kg straight Olympic bar and weights (75% 1RM_e_) in a controlled manner, flexing at the elbow, to the mid chest before immediately pushing upwards and extending at the elbows back to the starting position. Each repetition was self‐paced. Test failure was defined as the inability to complete a full repetition or a repetition with correct form as determined by a researcher.

The preacher curl is an isolated (biceps muscles) upper body exercise. Participants sat upright on the preacher seat with their elbows and forearms resting on the preacher pad. From the starting position (arms extended with hand grip at shoulder width), they flexed at the elbow to bring the 8.5 kg EZ bar and weights (75% 1RM_e_) toward themselves to achieve a 45° angle before eccentrically lowering the bar back to the starting position. Each repetition was self‐paced. Test failure was defined as inability to complete a full repetition or a repetition with correct form as determined by a researcher.

The squat jump is a lower body (quadriceps, glutes, and hamstring muscles) exercise. Participants stood with their feet shoulder width apart and knees slightly bent, flexed their knees to 90° maintaining a straight back and high chest. They extended their hip, knees and ankles and pushed up into a jump with minimal arm movement. Upon landing, they flexed their knees to 90° to absorb impact and returned to the starting position. The squat jumps were performed at a tempo of one repetition every 1.2 s that was externally paced using the sound of a metronome. Test failure was defined as inability to complete a repetition at the correct tempo or correct form as determined by a researcher.

### 2.4 Cognitive Tasks

The cognitive element of the BET program comprised a series of 3‐min cognitive tasks that were selected to activate executive function cognitive operations (e.g., Dallaway et al. 2024; Rautu et al. 2025). The four cognitive tasks (and their key executive functions) were 2‐back (updating), time‐load dual‐back (updating, inhibiting, switching), multi‐source interference (inhibiting), and switch stop visual (inhibiting). The tasks were performed using the SOMA‐NPT app (Soma Technologies, Switzerland) running on an iPhone. Please see https://intercom.help/soma‐technologies/en/articles/10449325‐cognitive‐tasks‐available‐inside‐soma‐npt for more details about the tasks. The cognitive tasks operated in adaptive individualized mode. The algorithm computed the number of correct responses in each block of 10 trials. If the number of correct responses in the just completed block of trials was 0–8, the mean interstimulus interval in the next block of 10 trials was increased by 50–150 ms, thereby making the task easier. If the number of correct responses in the just completed block of trials was 9–10, the mean interstimulus interval in the next block of 10 trials was decreased by 50–150 ms, thereby making the task harder. The interstimulus intervals for the tasks ranged from 200 to 1500 ms (see Supporting Information S1). A range of interstimulus intervals was employed in each task to reduce stimulus predictability. The cognitive tasks completed by the BET group are described below.

The 2‐back task presented a series of random letters on the screen and participants had to identify if the new letter was the same as the one that appeared two letters back. Participants indicated if the letter was the same as two letters back by pressing the left button and indicated that it was different by pressing the right button. The time‐load dual‐back task switched between a primary memory updating task and a secondary decision‐making task. In the 1‐back task, participants were presented with a letter on screen and responded as quickly and accurately as possible by tapping the left or right arrow buttons if the letter was or was not the same as the previous letter. In the decision‐making task, they were presented with odd or even numbers on screen and responded as quickly and accurately as possible by tapping the one button or two button, respectively. The multi‐source interference task required identification of the correct response despite distracting and irrelevant information. Participants were presented with three numbers (1, 2, 3) in the middle of the screen in one or two sizes (small and/or large). One of the numbers was presented twice and one of the numbers was presented once (the target). They responded by pressing as fast and as accurately as possible one of three buttons (1, 2, 3) at the bottom of the screen corresponding to the target number. The switch stop visual task presented a series of arrows that appeared either in left‐facing or right‐facing directions and participants responded by clicking the left or right button. They were instructed that if the color of the arrow changed from blue to red they should not press any buttons.

### 2.5 Procedure

#### 2.5.1 Testing

Participants completed two laboratory‐based testing sessions in weeks 1 and 6. After completing a standardized warmup (cycling for 6‐min at their preferred self‐selected intensity followed by six mobility and activation exercises; see Supporting Information S1), they performed the following sequence: 10 chest press repetitions at 75% 1RM_e_, RPE rating, 3‐min rest, chest press repetitions to failure at 75% 1RM_e_, 3‐min rest, 10 preacher curl repetitions at 75% 1RM_e_, RPE rating, 3‐min rest, preacher curl repetitions to failure at 75% 1RM_e_, 3‐min rest, squat jump repetitions to failure. After the initial 10 repetitions of the two resistance exercises, participants provided a rating of perceived exertion (RPE) using a Borg CR10 scale (Borg 1982), with anchors of 0 (*no effort at all*) and 10 (*maximal exerted effort*). They were told that they could not rest between repetitions. The number of repetitions to failure was recorded. Testing took place at the same time of day. Testing sessions were supervised by an experienced strength and conditioning personal trainer. At the end of the pre‐test session, participants received instruction and practiced the cognitive tasks that they performed in the training sessions.

#### 2.5.2 Training

Training took place in the participant's own gym. We opted for remote‐based training to give participants control, flexibility and autonomy over their training. The components of the 4‐week ET program are detailed in Supporting Information S1. All participants performed the same exercise tasks during training. Each week included one session based on push mechanics, one session based on pull mechanics, and one session based on combined push and pull mechanics plus core stabilization. Participants watched video tutorials and read written explanations about each exercise on the My PT Hub app.

Participants completed three gym‐based training sessions per week in weeks 2, 3, 4 and 5 (corresponding to training weeks 1–4, see Supporting Information S1). Sessions were separated by one or two rest days. The training program was delivered using the My PT Hub app (https://www.mypthub.net) running on an iPhone. At the start of each session, they completed a general warmup comprising 12‐min cycling followed by eight repetitions of six upper body activation and mobility exercises (neck stretches, arm circles, lateral side stretches, spinal stretches, hip crossovers, band pulls). For each training exercise, they performed three sets of six‐to‐eight repetitions, with each repetition comprising a controlled 3 s eccentric phase, an explosive 1 s concentric phase, and no pauses at the top or bottom. Participants rested for 2 min between sets (Froyd et al. 2013). The weight used in each exercise was selected (and adjusted) by the participant so that they could complete three sets of six (minimum) to eight (maximum) repetitions. This process indirectly allowed for progressive overloading. The training program was designed to develop muscular strength (Anderson and Kearney 1982).

Each week they performed sets of push exercises on day 1 (e.g., presses, raises, and extensions), sets of pull exercises on day 4 (e.g., rows, pullovers, curls), and sets of combined push and pull exercises plus sets of 30 s of core exercises on day 6 (e.g., crunches, sit‐ups, and planks). In training weeks 1–4, the BET group performed four 3‐min cognitive tasks during the 12‐min cycling warmup and one 3‐min cognitive task after the six mobility and activation exercises warmup (i.e., prior BET)—this amounted to 15‐min of cognitive tasks. In training weeks 1–2, the BET group performed five 3‐min cognitive tasks between the six exercises (i.e., intermixed BET), and one 3‐min cognitive task after the last exercise (i.e., post BET)—this amounted to 18‐min of cognitive tasks. In training weeks 3–4, the BET group performed three 3‐min cognitive tasks between the four exercises, and three 3‐min cognitive tasks after the last exercise—this amounted to 18‐min of cognitive tasks. In each training session across the 4 weeks, the BET group performed a total of 33‐min of cognitive tasks.

Participants in the active control (ET only) group used the SOMA‐NPT app to perform a box (paced) breathing task during the 3‐min periods instead of the executive function cognitive tasks. In this task, they were visually cued to breathe in through their nose for 4 s, hold their breath for 4 s, breathe out through their mouth for 4 s, and hold their lungs empty for 4 s. In each training session, the ET group performed a total of 33‐min of box breathing. We opted to have an active control group who used the SOMA‐NPT app to ensure that the use of a cognitive training app for the experimental group did not selectively influence exercise behavior.

### 2.6 Statistical Analyses

Statistical analyses were conducted using SPSS software (version 29). Significance level was set at *p* = 0.05. Preliminary analyses confirmed that there were no significant group differences in age, *t*(20) = 0.81, *p* = 0.43, body mass, *t*(20) = 0.26, *p* = 0.80, and sex, *χ* ^2^(1) = 0.11, *p* = 0.75. Exploratory analyses confirmed that the data met the assumptions of ANOVA. The primary analyses were a series of 2 group (BET, ET) by 2 sex (men, women) by 2 test (pre, post) ANOVAs were conducted on the number of repetitions to failure and ratings of perceived exertion. We included sex as a factor in these analyses because exercise endurance performance and perception of exertion sometimes differ between men and women, however, none of the interactions involving this factor significant. The reported analyses focused on the key group and test effects. Planned comparisons (ANOVAs) were used to examine pre‐post changes separately for each group and between groups. Partial eta‐squared (*η*_*p*_^2^) was reported as the effect size (Cohen 1992).

## 3 Results

### 3.1 Muscular Endurance Performance

**FIGURE 1:**
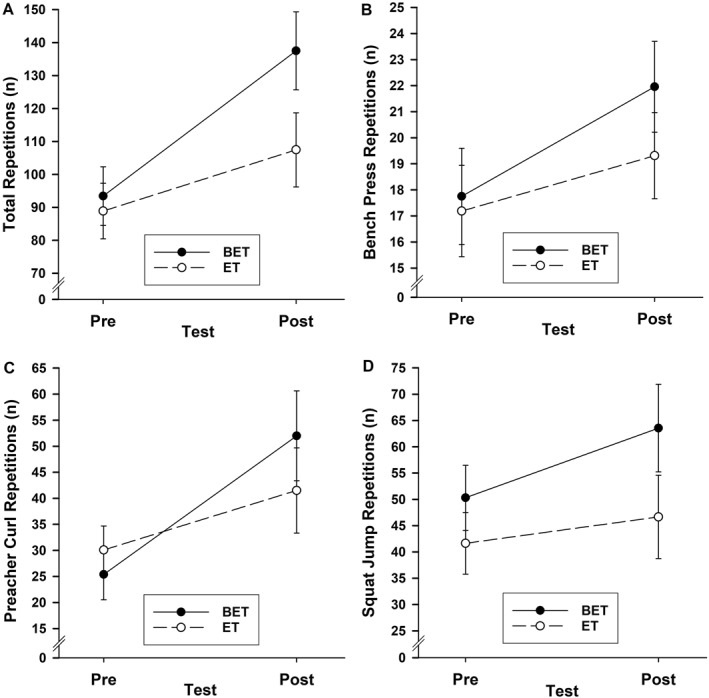
Mean (standard error) total (A), bench press (B), preacher curl (C) and squat jump (D) repetitions to failure as a function of group and test. BET = brain endurance training group; ET = exercise training group.

The number of exercise repetitions to failure increased from before to after completing 4 weeks of training, with the extent of the increase greater for BET than ET (Figure 1). A series of 2 group by 2 sex by 2 test ANOVAs yielded large main test effects for total, *F*(1, 18) = 48.53, *p* = 0.001, *η*_*p*_^2^ = 0.73, bench press, *F*(1, 18) = 12.91, *p* = 0.002, *η*_*p*_^2^ = 0.42, preacher curl, *F*(1, 18) = 16.57, *p* = 0.001, *η*_*p*_^2^ = 0.48, and squat jump, *F*(1, 18) = 21.71, *p* = 0.001, *η*_*p*_^2^ = 0.55, repetitions to failure. The group by test interaction effects were large for the total number of repetitions, *F*(1, 18) = 8.04, *p* = 0.01, *η*_*p*_^2^ = 0.31, medium for squat jump, *F*(1, 18) = 4.12, *p* = 0.05, *η*_*p*_^2^ = 0.20, medium for preacher curl, *F*(1, 18) = 2.64, *p* = 0.12, *η*_*p*_^2^ = 0.13, and small for bench press, *F*(1, 18) = 1.40, *p* = 0.25, *η*_*p*_^2^ = 0.07. Separate ANOVAs for each group indicated that the BET group completed more repetitions during the post‐test than the pre‐test for total (*p* = 0.001, *η*_*p*_^2^ = 0.72, Δ = +50%), bench press (*p* = 0.004, *η*_*p*_^2^ = 0.38, Δ = +33%), preacher curl (*p* = 0.001, *η*_*p*_^2^ = 0.46, Δ = +93%), and squat jump (*p* = 0.001, *η*_*p*_^2^ = 0.55, Δ = +28%), repetitions, whereas the exercise group completed more total repetitions post‐test compared to pre‐test (*p* = 0.008, *η*_*p*_^2^ = 0.33, Δ = +13%), but demonstrated no pre‐post improvements for bench press (*p* = 0.10, *η*_*p*_^2^ = 0.15, Δ = +13%), preacher curl (*p* = 0.09, *η*_*p*_^2^ = 0.15, Δ = +30%), and squat jump (*p* = 0.08, *η*_*p*_^2^ = 0.16, Δ = +12%) exercises. Analyses on the pre‐test to post‐test change scores confirmed that the BET group improved more than the exercise group for total (*p* = 0.01, *η*_*p*_^2^ = 0.28) and squat jump (*p* = 0.05, *η*_*p*_^2^ = 0.20) repetitions but not bench press (*p* = 0.25, *η*_*p*_^2^ = 0.07) and preacher curl (*p* = 0.12, *η*_*p*_^2^ = 0.13) repetitions. There were no group main effects.

### 3.2 Perceived Exertion

**FIGURE 2:**
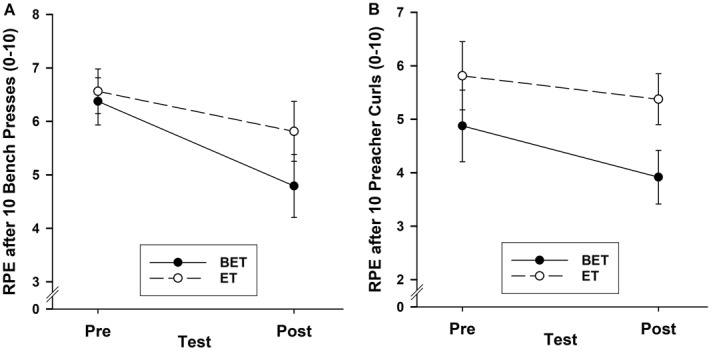
Mean (standard error) ratings of perceived exertion (RPE) after 10 bench press repetitions (A) and ten preacher curl repetitions (B) as a function of group and test. BET = brain endurance training group; ET = exercise training group.

The ratings of perceived exertion following completion of 10 repetitions of the resistance exercises decreased after 4 weeks of training, with the extent of the decrease somewhat larger for BET than ET (Figure 2). ANOVAs (2 group by 2 sex by 2 test) yielded medium test main effects for both bench press, *F*(1, 18) = 5.32, *p* = 0.03, *η*_*p*_^2^ = 0.23, and preacher curl, *F*(1, 18) = 4.05, *p* = 0.06, *η*_*p*_^2^ = 0.18, exercises; ratings declined from pre‐test to post‐test. The group by test interaction effect was small for bench press, *F*(1, 18) = 0.68, *p* = 0.42, *η*_*p*_^2^ = 0.03, and preacher curl, *F*(1, 18) = 0.56, *p* = 0.46, *η*_*p*_^2^ = 0.03. Separate analyses for each group indicated that perceived exertion in the BET group decreased from pre‐test to post‐test for bench press (*p* = 0.04, *η*_*p*_^2^ = 0.21, Δ = −22%) but not preacher curl (*p* = 0.07, *η*_*p*_^2^ = 0.17, Δ = −12%), whereas perceived exertion in the exercise only training group did not change from pre‐test to post‐test for bench press (*p* = 0.30, *η*_*p*_^2^ = 0.06, Δ = −9%) or preacher curl (*p* = 0.37, *η*_*p*_^2^ = 0.04, Δ = −3%). Analyses on the pre‐test to post‐test change scores indicated that these changes in perceived exertion were similar for the BET group and exercise group for bench press (*p* = 0.42, *η*_*p*_^2^ = 0.04) and preacher curl (*p* = 0.46, *η*_*p*_^2^ = 0.03) exercises.

## 4 Discussion

This study compared the effects of 4 weeks of remote and gym‐based BET and ET on muscular endurance performance (number of repetitions to failure) and effort perception (RPE) during exercise. BET (vs. ET) increased the number of total repetitions summed across the three exercise tasks by 50% (vs. 13%), bench press repetitions by 33% (vs. 13%), preacher curl repetitions by 93% (vs. 30%), and squat jump repetitions by 28% (vs. 12%). Moreover, RPE after completing 10 bench presses decreased by −22% following BET (vs. −9% for ET) whereas RPE after completing 10 preacher curls decreased by −12% following BET (vs. −3% for ET). Our key findings are considered below.

### 4.1 Endurance

Our first study purpose was to determine whether BET improves endurance exercise performance compared to standard ET. Participants in the BET group completed cognitive tasks before, between and after gym‐based upper body resistance and core exercises. The cognitive battery comprised brief versions of four classic executive function tasks that required updating, inhibiting, and switching cognitive operations (Dallaway et al. 2024; Rautu et al. 2025). In support of our hypothesis, we found that BET improved resistance and plyometric endurance exercise performance, indexed by the number of bench press repetitions at 75% 1RM_e_, preacher curl repetitions at 75% 1RM_e_ and squat jump repetitions until failure, compared to standard ET. The bench press findings (i.e., 33% improvement in BET group) exceed those observed in previous studies showing that BET improves repetitions to failure of bench press at 50% 1RM_e_ by 22% after 4 weeks (Rautu et al. 2025) and bench press at 34% 1RM_e_ by 10% after 3 weeks and 14% after 6 weeks (Díaz‐García et al. 2024) in experienced weightlifters. The preacher curl findings (i.e., 93% improvement in the BET group) exceed those reported in previous study showing that BET increased the number of bicep curls in a set period in inexperienced older adults by 7% after 4 weeks (Díaz‐García et al. 2025). The squat jump findings (i.e., 28% improvement in the BET group) are similar to or exceed those of previous studies showing that BET improves performance of squat jumps in experienced young adult weightlifters by 9% after 3 weeks and by 12% after 6 weeks (Díaz‐García et al. 2024), squat jumps in inexperienced young adults by 25% after 4 weeks (Dallaway et al. 2024), and squats in inexperienced older adults by 14% after 4 weeks (Díaz‐García et al. 2025). The extent of the performance improvements, which averaged 62% for preacher curl, 22% for bench press, and 20% for squat jumps, were greater for preacher curl than bench press and squat jump. We attribute this difference to preacher curls being a pure isolation movement that eliminates momentum, maximizes tension, and allows for superior, consistent technical execution. Nonetheless, it is worth noting that the relative improvement (computed as BET group change divided by ET group change), which were 3.1 for preacher curl, 2.5 for bench press and 2.3 for squat jumps, were broadly similar for the three exercises. This suggests that there was a common underlying process that preferentially benefited the physical performance of participants who completed combined cognitive and exercise training. A potential explanation for this performance benefit associated with BET is considered below.

### 4.2 Perceived Effort

Our second study purpose was to determine whether BET alters perception of effort while performing resistance exercises more than standard ET. In partial support of our hypothesis, we found that BET reduced effort perception by 22% during bench press exercise and by 12% during preacher curl exercise (albeit a tendency) whereas the standard training did not alter perception of effort during exercise (which fell by 9% and 3%, respectively). The observation that training benefited bench press more than preacher curl may be attributed to the motor control complexity requirements (i.e., effort) of the two lifting actions, with bench press involving several muscles and preacher curl involving just one muscle (Lagally et al. 2002). The finding that effort perception was lower during exercise following BET is compatible with evidence that BET reduced RPE during cycling in young adults who were highly trained cyclists (Staiano et al. 2023), trained cyclists (Barzegarpoor et al. 2021), and active adults with some cycling experience (S. M. Marcora et al. 2014). Taken together, these findings provide more evidence that the addition of demanding cognitive tasks to standard physical training programs can recalibrate the relationship between perception of effort and physical load. This evidence provides additional support for the predictions of the psychobiological model of exercise endurance (S. Marcora 2019).

### 4.3 Limitations and Future Directions

The current study confirmed the benefits of BET on the performance of resistance and plyometric exercise to failure. Nonetheless, it is important to consider potential study limitations when interpreting this endurance enhancement effect. First, the study tested moderately experienced weightlifters. The generalizability of our findings to highly experienced (or novice) weightlifters therefore needs to be determined. It is possible that training experience may moderate the effects of BET on endurance performance. This might be because trained weightlifters interpret the additional cognitive tasks as a challenge and thus be more engaged whereas untrained weightlifters interpret the additional cognitive tasks as a threat and thus be less engaged and overwhelmed. Therefore, future studies should evaluate the effects of BET on weightlifting‐based resistance and calisthenic‐based power exercises in different groups of novice and expert athletes. Second, 1RM was estimated rather than measured. Given the number of bench press repetitions to failure (Figure 1), and that participants performed 10 repetitions before attempting to push or pull weights to failure, it is likely that we underestimated their 1RM at the start of the study (Nuzzo 2024). Accordingly, it is likely that participants performed the bench press and preacher curl tests at a lower relative intensity (c. 60%–65% 1RM) rather than 75% 1RM. Future BET studies should measure 1RM directly (Rautu et al. 2025). Our findings still show that BET improved exercise endurance repetitions to failure, it is just that they were performed at a lower intensity than expected. Third, we tested endurance performance on three exercises—bench press, preacher curl and squat jump. It is likely that the effects of BET will differ among exercise tests because their requirements are different (e.g., Dallaway et al. 2024). For instance, the preacher curl is single joint isolated movement, the bench press is a multi‐joint compound movement, and the squat jump is a sequence of two multi‐joint compound movements. In the present study, the extent of the improvement in the number of repetitions was associated with the complexity of the exercise. Accordingly, BET can be expected to benefit complex actions more than simple actions. Such differences could be explored in programmatic research looking at a broad range of exercise test options. Fourth, we did not measure cognitive states, physiological responses or movement kinematics, such as flow, muscle activity or movement velocity (Mortimer and Ring 2025; Rautu et al. 2025), to evaluate mechanisms that might help explain the BET‐related enhancements in endurance exercise performance. Accordingly, future studies could determine the extent to which increases in the number of exercise repetitions with BET are mediated by improvements in cognitive states, muscle activation patterns and movement kinematics.

### 4.4 Practical Applications

Strength and conditioning coaches, personal trainers, professional athletes and recreational weightlifters wishing to improve muscular endurance are encouraged to explore the benefits of adding short demanding cognitive tasks to their standard physical training programs. This study shows that combined cognitive and ET is pragmatic in unsupervised settings. Individuals can easily perform these cognitive tasks during their gym sessions and track their performance and impact using apps running on personal phones and tablets. The current study provides an example of a training plan which shows how this can be implemented by athletes as part of their standard gym training regime. These apps have batteries of cognitive tasks that can offer variety to ensure that the cognitive demands can be calibrated and individualized to suit the individual's cognitive capacity and current cognitive state. They also offer flexibility to fit with any physical training program that comprises multiple exercises and sets, and that varies in duration. In sum, this sports technology offers a bespoke solution to recreational and professional athletes wishing to push themselves further during training and subsequently perform better when participating in organized sport where enhanced endurance confers a competitive advantage.

## 5 Conclusion

An app‐based remote‐based BET program enhanced endurance exercise performance in recreational weightlifters. Specifically, BET increased the total number of repetitions to failure of bench press, preacher curl and squat jump exercises more than ET alone, with the 50% improvement in the BET group almost four times better than the 13% improvement in the ET group. Training was associated with less perceived effort during resistance exercise, with the pre‐test to post‐test reduction tending to be more substantial following BET than ET, suggesting that BET elicited a stronger stimulus that recalibrated the sense of effort, which is key to endurance exercise performance in the psychobiological model (S. Marcora 2019). The current evidence extends the benefits of BET as a flexible and autonomous method for performance enhancement to resistance and plyometric exercise endurance domains in recreational weightlifters.

## Funding

The authors have nothing to report.

## Conflicts of Interest

The authors declare no conflicts of interest.

## Supporting information


Supporting Information S1

## Data Availability

The data that support the findings of this study are available from the corresponding author upon reasonable request.
