# Supplementary material for: Remote‐Based Brain Endurance Training Enhances Bench Press, Preacher Curl, and Jump Squat Performance in Recreational Athletes
Source: Eur J Sport Sci. 2026 Apr 13;26(5):e70173. doi: 10.1002/ejsc.70173 (PMC13076188; doi:10.1002/ejsc.70173)
Supplement: Supplementary file 1 — Supporting Information S1 [file EJSC-26-e70173-s001.docx]

**A 4-Week Gym Exercise Program**

***Warmup (weeks 1-4, sessions 1-3)***

12-min cycling plus upper body activation and mobility exercises (side neck stretch, arm circle, reaching lateral side stretch, spinal stretch, hip crossover, band pull apart)

***Push Exercises (weeks 1-2, session 1)***

barbell bench press, incline dumbbell chest press, standing barbell shoulder press, seated dumbbell lateral raise, narrow grip EZ bar skullcrusher, cable tricep push down bar attachment

***Pull Exercises (weeks 1-2, session 2)***

bent over barbell row, wide-grip EZ bar pullover, seated cable row, wide grip lat pulldown, wide grip EZ bar preacher curl, standing hammer curl

***Combination Exercises (weeks 1-2, session 3)***

landline single arm jammer, barbell good morning, dumbbell shrug, high cable fly

***Core Exercises (weeks 1-2, session 3)***

v-up, plank with rotation, toe touch, scissor kick, russian twist with medicine ball, reverse crunch

***Push Exercises (weeks 3-4, session 1)***

flat dumbbell chest press, seated Arnold press, narrow grip barbell bench press, kneeling cable tricep extension

***Pull Exercises (weeks 3-4, session 2)***

chest supported dumbbell row, facepull, cable lateral raise, single arm dumbbell preacher curl

***Combination Exercises (weeks 3-4, session 3)***

landmine 180, landmine single arm row, cable chest press, hyperextension

***Core Exercises (weeks 3-4, session 3)***

plank, sit-up, hanging leg raise, air bike, deadbug, russian twist with medicine ball

**Cognitive Tasks Performed by BET Group**

**2-Back Task**

A task where a series of random letters appeared on the screen and participants had to identify if the new letter was the same as the one that appeared 2 letters back. Participants indicated if the better was the same as 2 back by pressing the ‘left’ button and indicate that it was different by pressing the ‘right’ button. The interstimulus intervals ranged from 200-1000 ms.

**Time Load Dual Back Task**

A task where a series of random letters and numbers (between 1 and 9) were presented in turn. If the letter was the same as the previous letter, participants pressed the ‘left’ button, if the number presented was odd participants pressed the ‘1’ button and if the number was even, participants pressed the ‘2’ button. The interstimulus intervals ranged from 200-1000 ms.

**Multisource Interference Task**

A task where three numbers appeared on the screen in different size fonts, one number appeared twice and one was different. Participants were required to identify the ‘target number’ and press the corresponding button (e.g. if 131 appeared, 3 was the target number as participants should press the 3 button). The interstimulus intervals ranged from 400-600 ms.

**Switch Stop Visual Task**

A task where a series of arrows appeared either in a left or right directions and participants must respond by clicking the left or right button. If the color of the arrow changed from blue to red, the participants were told to resist pressing any buttons. The interstimulus intervals ranged from 250-1500 ms.
